# Exploring the Mechanisms Underlying Cellular Uptake and Activation of Dendritic Cells by the GK-1 Peptide

**DOI:** 10.1021/acsomega.4c07736

**Published:** 2024-11-28

**Authors:** Jacquelynne Cervantes-Torres, Juan A. Hernández-Aceves, Julián A. Gajón Martínez, Diego Moctezuma-Rocha, Ricardo Vázquez Ramírez, Sergio Sifontes-Rodríguez, Gemma L. Ramírez-Salinas, Luis Mendoza Sierra, Laura Bonifaz Alfonzo, Edda Sciutto, Gladis Fragoso

**Affiliations:** 1 Departamento de Inmunología, Instituto de Investigaciones Biomédicas, Universidad Nacional Autónoma de México, Ciudad de México MX 04510, Mexico; 2 Departamento de Microbiología e Inmunología, Facultad de Medicina Veterinaria y Zootecnia, Universidad Nacional Autónoma de México, Ciudad de México MX 04510, Mexico; 3 Unidad de Investigación Médica en Inmunoquímica, Hospital de Especialidades, CMN Siglo XXI, Instituto Mexicano del Seguro Social, Ciudad de México MX 06600, Mexico; 4 Departamento de Biología Molecular y Biotecnología, Instituto de Investigaciones Biomédicas, Universidad Nacional Autónoma de México, Sede Tercer Circuito Exterior Edificio C 1er Piso, C-146, Ciudad de México MX 04510, Mexico; 5 Investigador por México del CONAHCyT adscrito al Departamento de Inmunología, Instituto de Investigaciones Biomédicas, Universidad Nacional Autónoma de México, Sede Circuito Escolar Edificio A 1er Piso, Ciudad de México MX 04510, Mexico

## Abstract

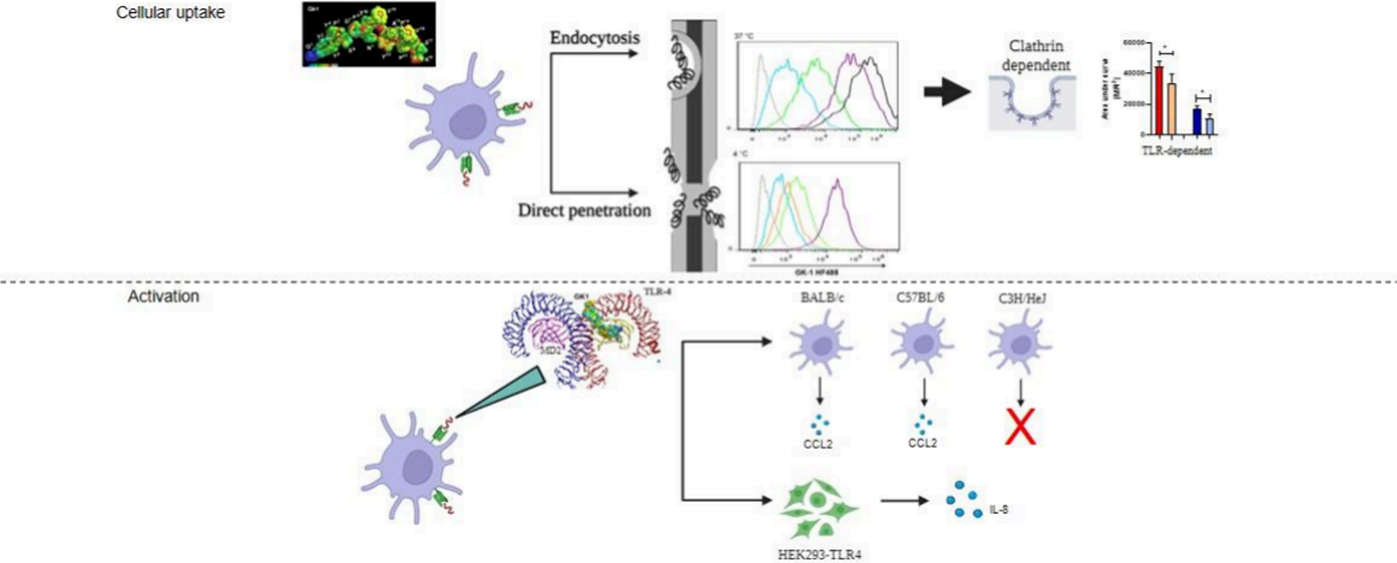

The use of peptides for cancer immunotherapy is a promising and emerging approach that is being intensively explored worldwide. One such peptide, GK-1, has been shown to delay the growth of triple-negative breast tumors in mice, reduce their metastatic capacity, and reverse the intratumor immunosuppression that characterizes this model. Herein, it is demonstrated that GK-1 is taken up by bone marrow dendritic cells in a dose-dependent manner 15 min after exposure, more efficiently at 37 °C than at 4 °C, implying an entrance into the cells by energy-independent and -dependent processes through clathrin-mediated endocytosis. Theoretical predictions support the binding of GK-1 to the hydrophobic pocket of MD2, preventing it from bridging TLR4, thereby promoting receptor dimerization and cell activation. GK-1 can effectively activate cells via a TLR4-dependent pathway based on *in vitro* studies using HEK293 and HEK293-TLR4-MD2 cells and *in vivo* using C3H/HeJ mice (hyporesponsive to LPS). In conclusion, GK-1 enters the cells by passive diffusion and by activation of the transmembrane Toll-like receptor 4 triggering cell activation, which could be involved in the GK-1 antitumor properties.

## 1 Introduction

Immunotherapy has revolutionized cancer treatment, adding clear clinical benefits to standard approaches (chemotherapy, radiotherapy, and surgery) and improving patient survival and quality of life in several cancer types.^1^ This intervention is based on the manipulation of patient immunity to reverse the cellular depletion and/or suppressive environment prevailing in the tumor microenvironment, mainly through immune checkpoint blockade and chimeric antigen receptor T-cell therapies.^2^

GK-1, an 18-amino acid peptide, is a promising candidate for cancer immunotherapy, either alone or in combination with other therapies. Peritumoral or intravenous GK-1 injections significantly reduced tumor growth and increased survival in a murine melanoma model.^3,4^ GK-1 also increased survival and significantly reduced lung macro-metastases in the 4T1 model of breast cancer in BALB/c mice.^5^ In both cancer models, GK-1 significantly reduced PD-1 expression in tumor-infiltrating CD8 T cells, leading to increased production of effector molecules such as IFN-γ and granzyme B.^4,6^ It is relevant to compare the potential use of GK-1 vs immunotherapy based on the use of monoclonal antibodies against PD-1 and PD-L1, the most widely immune checkpoint inhibitors employed worldwide to fight against different cancers. The latter is based on the rupture of the peripheral tolerance resulting in the restoring of T-cell activity and the increasing of autoimmune-related severe side effects.^7^ Meanwhile, as we previously demonstrated, GK-1 did not exhibit any toxicity or mutagenic activity even used at doses up to 12.5 mg/kg.^8^ Another point, no less relevant, is the enormous difference in therapy costs between immune checkpoint inhibitors and the GK-1 peptide.

Understanding the cellular uptake of GK-1 and how it triggers intracellular signaling will contribute to elucidating the potential mechanisms underlying its antitumor properties. Among the cell types activated by GK-1 are antigen-presenting DCs and macrophages (Mφ). The latter play a role in multiple functional activities, not only in immunity, but also in the regulation of lipid and iron metabolism and wound healing.^9,10^ Activated Mφ are required to eliminate invading microorganisms and tumor cells. Activation of Mφ involves a series of morphological and biochemical changes that promote diverse functions.^11^ GK-1 causes classical activation of peritoneal Mφ, enhancing their phagocytic capacity, along with increased production of the chemokines CCL2 and CCL3, the proinflammatory cytokine IL-6, and nitric oxide.^12^ In addition, GK-1 activates bone marrow-derived dendritic cells (BM-DCs) by increasing the expression of MHC class II and CD86 via activation of the MAPK and NF-κB pathways.^13^ These effects on DCs are of particular interest as they are key players in the initiation of adaptive immune responses.^14^ Indeed, DCs are currently used in immunotherapy of cancer and infectious diseases.^15^ These effects of GK-1 could lead to enhanced immunity against tumorigenic cells.

On the other hand, our group has reported that GK-1 induces the activation and effector activity of antigen-presenting cells (APCs) through a Myd88-dependent mechanism, with a Toll-like receptor as a possible target.^13^ Here, the mechanisms involved in the cellular uptake of GK-1 and the subsequent cell activation were investigated both theoretically and experimentally.

## 2 Materials and Methods

### 2.1 Uptake Studies

#### 2.1.1 Mouse Bone Marrow-Derived Dendritic Cells

Cell suspensions were obtained from femurs and tibias of C57BL/6J mice and cultured for 6 days in RPMI 1640 medium supplemented with 50 μM 2-mercaptoethanol (Gibco, Thermo Fisher Scientific, Waltham, Massachusetts, USA), 10 mM HEPES (Promega, Madison, Wisconsin, USA), 20 μg/mL gentamicin, 5% (v/v) fetal bovine serum (FBS, Gibco), and 20 ng/mL recombinant murine granulocyte-macrophage colony-stimulating factor (GM-CSF) (PreproTech, Cranbury, New Jersey, USA). On day 3 of culture, the medium was replaced with a fresh complete medium containing GM-CSF. Cells were separated from the monolayer on day 6, as previously described.^13^

#### 2.1.2 Cellular Uptake Studies

For cellular uptake assays, 10^6^ BM-DCs were incubated in sterile 5 mL tubes at different temperatures (4 or 37 °C), incubation times (5 min to 5 h), and concentrations (1, 2.5, 5, 7.5, 10, 50, or 100 μM) of HiLyte Fluor 488-labeled GK-1 (GK1-HF488) (AnaSpec, Fremont, California, USA) as indicated in the figure legends. After incubation, the cells were washed and treated with trypsin (1 mg/mL) for 15 min at 37 °C and then washed twice with phosphate-buffered saline (PBS). BM-DCs were then blocked with anti-FcR antibody (CD16/32) for 20 min at 4 °C in 20 μL of PBS supplemented with 5% FBS and 0.02% sodium azide (staining buffer) and then stained with CD11c-PE-Cy5.5 (N418, BioLegend, San Diego, California, USA). The cells were then washed with PBS (1200 rpm, 5 min) and immediately acquired in an Attune Nxt cytometer (Thermo Fisher Scientific) and analyzed using the FlowJo software (v.8.7, FlowJo LLC, Ashland, Oregon, USA).

#### 2.1.3 Calcein Release Assay

BM-DCs were stained with calcein to rule out pore formation by GK-1. Briefly, 10^6^ cells were resuspended in 2 μM calcein AM staining media (Life Technologies) and incubated for 30 min at 37 °C under 5% CO_2_. Then, the cells were washed and incubated for 4 h in RPMI 1640 containing 10, 50, or 100 μM GK-1. Maximum calcein release was measured by incubating BM-DCs in 1% (v/v) Triton X-100 for 5 min. Spontaneous release (background) was determined by incubating BM-DCs without GK-1.

Cytotoxicity, expressed as the percent calcein release, was calculated for each sample using the formula [(percentage of BM-DC fluorescent cells in spontaneous release – percentage of BM-DC fluorescent cells in test)/(BM-DC fluorescent cells in spontaneous release)] × 100. All assays were performed in triplicate. Fluorescence intensity data from image cytometry was analyzed by using FlowJo.

#### 2.1.4 Cytotoxicity of GK-1 on BM-DCs

The *in vitro* cytotoxicity assay of GK-1 on BM-DCs was performed as described elsewhere^16^ with minor modifications. Briefly, 200 μL/well of a BM-DC suspension adjusted to 10^5^ cells/mL was seeded into 96-well culture plates (Nunc Corning, Thermo Fisher Scientific) in Dulbecco’s modified Eagle medium (DMEM) supplemented with 10% FBS and the GK-1 peptide at two-fold serial concentrations ranging from 16 to 500 μM. Each peptide concentration was tested in triplicate. BM-DC cultures in medium containing 10% dimethyl sulfoxide (DMSO, Sigma-Aldrich, Burlington, Massachusetts, USA) were included as a positive control of cytotoxicity. After 48 h of incubation at 37 °C under 5% CO_2_, 20 μL per well of 2 mM resazurin (Invitrogen, Waltham, Massachusetts, USA) in 0.9% NaCl solution was added. Relative fluorescence intensity (550 nm excitation, 590 nm emission, 50% gain) was read after overnight incubation using a Cytation 3 Epoch fluorescence plate reader (Agilent, Santa Clara, California, USA). Viability at each GK-1 concentration was calculated using the following formula: viability (%) = 100 × (Fx – Fmin)/(Fmax – Fmin), where Fx is the fluorescence intensity at a given GK-1 concentration, Fmin is the average fluorescence intensity of positive control cultures of cytotoxicity, and Fmax is the average fluorescence intensity of nontreated negative control cultures.

#### 2.1.5 Endocytosis Inhibition Studies

To evaluate endocytosis inhibitors, the cells were preincubated with the inhibitors for 30 min at 37 °C, washed with PBS, centrifuged at 1200 rpm for 5 min, and incubated with 10 μM GK-1-HF488 for 1 h at 37 °C. Finally, BM-DCs were stained and acquired on an Attune Nxt cytometer as described above.

The cells were treated with either sodium azide (energy-dependent endocytosis inhibitor),^17^ nystatin (caveolin-mediated endocytosis inhibitor),^18^ cytochalasin D (actin polymerization inhibitor),^19^ ethylenediaminetetraacetic acid (EDTA, calcium depletion),^20^ or potassium-depleted buffer in hyperosmotic sucrose solution (isotonic K^+^-free buffer containing 450 mM sucrose, clathrin-mediated endocytosis inhibitor).^21^ All reagents were purchased from Sigma-Aldrich.

#### 2.1.6 Confocal Microscopy

BM-DCs were seeded at 2 × 10^5^ cells/well in a four-well chamber slide (Lab-Tek II Chamber Slide, NUNC, Thermo Fisher Scientific) and incubated at 37 °C for 24 h. Then, they were incubated for the indicated time at 37 °C in RPMI containing a 10 μM solution of the labeled peptide. The cells were washed three times with PBS and stained with biotinylated CD11c (BioLegend) and streptavidin-Texas Red (Invitrogen) for 20 min to visualize DCs. Finally, the cells were washed, mounted on a glass slide, and processed in Vectashield mounting medium with 4′,6-diamidino-2-phenylindole (DAPI, Vector Laboratories, Newark, California, USA). The cells were photographed under a 40× objective on a Zeiss LSM confocal microscope. Z-stack images were visualized at a 10 μm depth.

#### 2.1.7 Statistical Analysis

All experiments were performed in triplicate. For flow cytometry assays, 10 000 events were acquired for each sample. CD11c+ cells were selected, and the gated region was used to assess peptide internalization, expressed as mean fluorescence intensity (MFI). MFI was corrected with respect to the unlabeled GK-1 peptide (GK-1 without a HiLyte Fluor marker) for each assay. The normality of data was tested by the Shapiro–Wilk test. Data were compared by one-way ANOVA followed by Dunnett’s multiple comparison test or Tukey’s multiple comparison test, or by unpaired Student’s *t* test using GraphPad Prism v. 5.03. Data are presented as the mean ± standard error of the mean (SEM); *p* < 0.05 indicates statistically significant differences.

### 2.2 *In Silico* Analysis of GK-1 as a Ligand for TLR4/MD2

#### 2.2.1 Molecular Structure of the Receptor, the GK-1 Peptide, and Lipopolysaccharide (LPS)

The structure of human tetrameric TLR4/MD2 in complex with two LPS sequences (Ra *Escherichia coli* chemotype) was obtained from the Protein Data Bank;^22^ PDB ID: 3FXI.^23^ The crystal structure of the complex consisted of two TLR4 polypeptide chains (TLR4-A and TLR4-B) and two MD2 chains (MD2-C and MD2-D). Water and saccharide molecules were removed from the LPS. To compare the dimers, the backbone overlap and root mean square deviation (RMSD) of TLR4-A with TLR4-B were evaluated for residues E27-C627; MD2-C was compared with MD2-D for Q19-N158, using the Superpose web server.^24^

The three-dimensional (3D) structure of GK-1 (G_1_YYYP_5_SDP_8_NTFYAP_14_PYSA_18_) was obtained using the peptide structure prediction server Pep Fold 3.^25^ The total energy in five conformations predicted by Pep Fold 3 was calculated, and the conformation of GK-1 with the lowest total energy was used for the docking analysis. LPS was used as a control molecule for *in silico* analysis. The structure of LPS was obtained directly from the cocrystallization structure of the TLR4/MD2-LPS complex, PDB ID: 3FXI. Total energy and electronic and physicochemical properties of GK-1 and LPS were calculated with a single point using the ωB97X-D density functional and the 6-31G* basis set with Spartan’20 software.^26^ Finally, the APBS biomolecular solvation software suite APBS^27^ was used to assign protonation states to receptor residues and ligands under biological conditions (pH 7.2).

#### 2.2.2 Molecular Docking

The 3D crystal structure of the TLR4/MD2 complex was used as the initial input to perform molecular docking and molecular dynamics analyses. LPS, water molecules, and redundant ligands, including glycosyl groups, were removed from the TLR4/MD2 complex. The molecular structures of GK-1 and TLR4/MD2 were converted to the PDBQT format for docking calculations using AutoDock Tools v.1.5.6.^28^ All rotatable bonds in GK-1 were left free, while the receptor was kept rigid and the polar hydrogen and Gasteiger charges were assigned. In a docking setup, grid dimensions were 82, 114, and 126, with the following coordinates: *x* = 12, *y* = −8, *z* = −6. The spacing box was 1.0 Å, and exhaustiveness was set to 300. Molecular docking was performed using AutoDock Vina 1.1.2.^29^

#### 2.2.3 Molecular Dynamics Simulations (MDS) of the TLR4/MD2-GK-1 Complex

For MDS, the tetramer (TLR4-A/MD2-C and TLR4-B/MD2-D) was used without accessory ligands or water molecules. The GK-1 conformation resulting from the docking analysis was positioned at the TLR4-A/MD2-C binding site using Discovery Studio (DS) software.^30^ The TLR4/MD2-GK-1 complex was placed in the center of a cube (160.3 × 157.7 × 158.1 Å = 4.0 × 106 Å^3^) filled with water molecules and ions. The protonation states of the ionizable residues (Asp, Glu, Lys, Arg, and His) were assigned to mimic a pH 7 environment. The total charge of the system, consisting of 19 e–, was neutralized by adding Na^+^ ions that randomly replaced water molecules to mimic a realistic ionic strength.

Initially, to prevent atomic collisions, energy minimization was performed using the steepest descent algorithm, with a step size of 0.002 kJ mol^–1^. The protein–solvent system was optimized for position-restricted equilibration under a canonical (NVT—number of particles, volume, and temperature) ensemble for 100 ps, and then an isothermal–isobaric (NPT—number of particles, pressure, and temperature) ensemble for 500 ps. The linear constraint solver (LINCS) was used to restrict covalent bonds.^31^ Long-range interactions were calculated using particle mesh Ewald (PME),^32^ with a short-range electrostatic cutoff and a short-range van der Waals cutoff of 1.2 Å. For the Coulomb and Lennard-Jones interactions, a cutoff of 1.0 nm was used. For the NPT ensemble, the temperature was set to 300 K with the Nosé–Hoover thermostat,^33^ and the pressure was set to 1 bar with the Parrinello–Rahman barostat.^34^ The systems used periodic boundary conditions in all three directions. Finally, unconstrained MDS was performed with the same NPT equilibrium parameters as above. MDS was performed for the TLR4/MD2-GK-1 complex for 50 ns. In the cluster analysis of trajectories, the RMSD was used to obtain a representative structure of the TLR4/MD2-GK-1 complex resulting from the sampling. The last 20 ns of stable trajectories in the protein backbone atoms were used for clustering analysis, with an RMSD cutoff of 3 Å. The trajectory file consisted of one frame saved every 10 ps for analysis. All MDS were performed using the Charmm27 force field^35^ and Gromacs 2022.2 software.^36^ The trajectories were analyzed with the cluster1 tool of Gromacs, and the visualizations were done with the VMD software.^37^

#### 2.2.4 *In Silico* Interaction Analysis of the TLR4/MD2-GK-1 and TLR4/MD2-LPS Complexes

The free energy of interaction of GK-1-receptor complexes (GK-1 with TLR4-A/MD2-C-TLR4-B/MD2-D) was calculated by *ab initio* methods. The representative conformation results from the last 20 ns of MDS for the GK-1-receptor and the LPS cocrystallization structure were analyzed.

In the GK-1-TLR4/MD2 and LPS-TLR4/MD2 complexes, the amino acid residues and molecular interactions involved were identified using the DS software. Only the first core of residues in the TLR4/MD2 binding pocket was included, and the cutoff distance between the ligand (GK-1 and LPS) and the tetrameric receptor was set to 3.4 Å for hydrogen bonds and 4.5 Å for hydrophobic interactions. The free energies of interaction included the ligand molecule and all adjacent binding pocket residues of the receptor. The following formula provided a simplified procedure for calculating the interaction energy (IE): IE = [RL] [*R* + *L*], where IE is the binding interaction energy, RL is the energy of the complex formed by the receptor residues and the ligand, *R* is the energy of the TLR4/MD2 residues, and *L* is the energy of the ligand. Binding interaction energies were calculated by single-point calculations using density functional theory (DFT) with the ωB97X-D function and the 6-31G* basis set level (for a detailed description of all *ab initio* methods used in this work, see ref (38)). All *in silico* procedures were performed on a 16-core computer with a 3.4 GHz Xeon processor.

### 2.3 Evaluating TLR4 Activation by GK-1

#### 2.3.1 Peptide

The GK-1 peptide (GYYYPSDPNTFYAPPYSA) was synthetically produced under Good Manufacturing Practices (98% purity, endotoxin-free) by USV Ltd., Mumbai, India. The peptide was dissolved in 0.9% saline solution at the concentration required for each assay.

#### 2.3.2 Cell Culture

The HEK293-null cell line (ATCC, CRL 1573, Manassas, Virginia, USA) was cultured in DMEM supplemented with 10% FBS and 1% penicillin and streptomycin. The HEK293-hTLR2/TLR6 cell line (InvivoGen, San Diego, California, USA, cat. 293-htlr2/6), HEK293-hTLR3 cell line (InvivoGen, cat. 293-htlr3), HEK293-hTLR5 cell line (InvivoGen cat. 293-htlr5), and HEK-293XL-hTLR7 cell line (InvivoGen cat. 293xl-htlr7) were cultured in DMEM with 10% FBS plus 10 μg/mL blasticidin. The HEK293-hTLR4-MD2-CD14 (HEK293-hTLR4) cell line (InvivoGen, cat. 293-hmd2cd14) was cultured in DMEM supplemented with 10% FBS plus 10 μg/mL blasticidin and 50 μg/mL hygromycin B. All of these cells were cultured according to the manufacturer’s instructions. HEK293-hTLR4, HEK293-hTLR2/TLR6, HEK293-hTLR3, HEK293-hTLR5, and HEK-293XL-hTLR7 cells require blasticidin to express pUNO, and HEK293-hTLR4 requires hygromycin B to express pDUO. The cells produce the cytokines IL-8, IL-6, and TNF-α when activated by the respective TLR ligands. IL-8 was used to detect cell activation through NF-κB because it is the most stable cytokine over time.^39,40^ The cells were cultured at 37 °C under 5% CO_2_ until >85% confluence was reached. Cells were then detached from the plates using PBS with 0.5 mM EDTA for 3 min.

#### 2.3.3 Activation Assay

Cell viability was assessed by trypan blue exclusion. Then, 2 × 10^5^ cells were stimulated for 16 h with either GK-1 at concentrations ranging from 5 to 150 μM or the appropriate TLR’s ligand: TLR2/TLR6–lipoteichoic acid (LTA, 10 μg/mL), TLR3–polyinosinic:polycytidylic acid (PolyI:C, 10 μg/mL), TLR5–flagellin (10 μg/mL), TLR7–CL264 (InvivoGen cat. tlrl-c264e-c, 10 μg/mL), or TLR4–lipopolysaccharide (LPS, 0.1 μg/mL, equivalent to 66 μM). Culture supernatants were collected, and IL-8 was quantified with an ELISA kit (BioLegend Cat. 431504), following the manufacturer’s protocol.

#### 2.3.4 Generating and Activating Bone Marrow Dendritic Cells by Treatment with GK-1 or LPS

Bone marrow cells were obtained from the femurs and tibias of BALB/c, C57BL/6, and C3H/HeJ mice (the latter carrying a mutation in the TLR4 gene). Erythrocytes were removed by incubation with a lysis solution (NH_4_Cl/KHCO_3_/EDTA). The cells were then suspended at a concentration of 10^6^ cells/mL in RPMI 1640 medium (supplemented with 50 μM 2-mercaptoethanol, 10 mM HEPES, gentamicin at 20 μg/mL, 5% (v/v) FBS, and 20 ng/mL recombinant GM-CSF) in non-adherent plates (Corning Cat. 3474). On day 3 of culture, the medium was replaced with fresh medium plus recombinant GM-CSF to induce differentiation into BM-DCs. On day 7, BM-DCs were harvested with >70% purity, as measured by CD11c+/IaIe+ expression. BM-DCs were treated with GK-1 at concentrations ranging from 10 to 300 μg/mL or with 10 ng/mL LPS, with 10 μg/mL Pam3CSK4, or left untreated. 24 h later, culture supernatants were collected, and CCL2 levels were quantified using an ELISA kit (BioLegend Cat. 432704) according to the manufacturer’s instructions.

### 2.4 Detection of GK-1 within HEK293-Null and HEK293-TLR4 Cells Cultured at 4 and 37 °C

HEK293 and HEK293-TLR4 cells were cultured as described above. Briefly, 5 × 10^5^ cells/well were cultured in U-bottom, 96-well plates. GK-1-HF488 was added at 0, 5, 10, and 50 μM, in triplicate for each cell line. One plate was incubated with both cell lines at 4 °C for 1 h, and another was incubated at 37 °C for 1 h. The cells were then detached from the wells and treated with trypsin (1 mg/mL) for 15 min at 37 °C. Then, they were washed with DMEM supplemented with 10% FBS and fixed with 2% paraformaldehyde. The cells were analyzed using a NxT flow cytometer. MFI was used to measure the amount of GK-1 internalized by the cells.

#### 2.4.1 Statistical Analysis

IL-8 levels were compared by two-way ANOVA. Sidak multiple comparisons were used, and Tukey multiple comparisons were used for CCL2 production. Statistical analysis was performed using GraphPad Prism v.8.00 for Windows. Data are expressed as mean ± SEM and were considered statistically significant at *p* < 0.05.

## 3 Results

### 3.1 GK-1 Enters BM-DCs via Clathrin-Mediated Endocytosis

**Figure 1:**
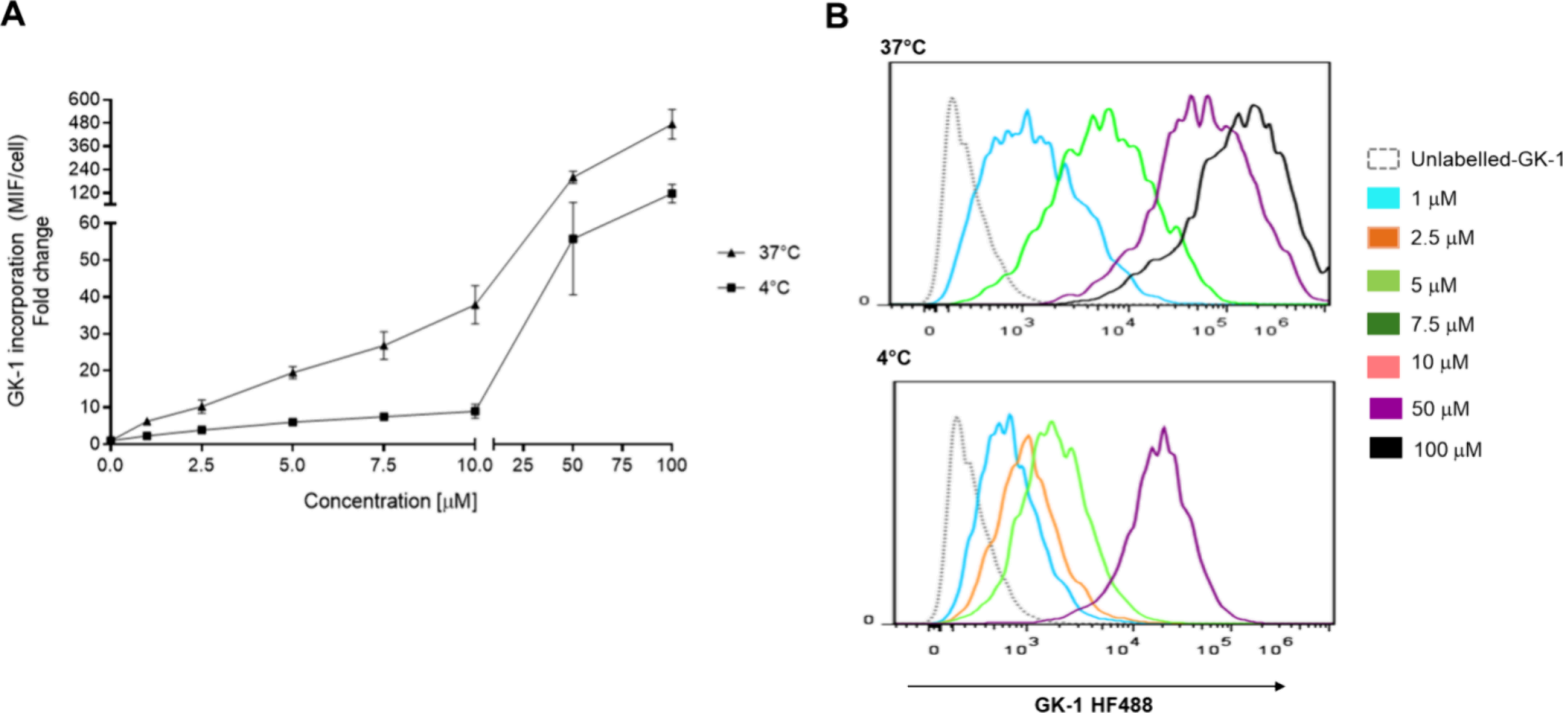
Kinetics of GK-1 uptake at 37 and 4 °C. (A) BM-DCs were incubated for 1 h in the presence of 1, 2.5, 5, 7.5, 10, 50, or 100 μM GK-1 coupled to HiLyte Fluor 488 at 4 °C (squares) or 37 °C (triangles). Samples were trypsinized before staining with anti-CD11c antibody and analyzed by flow cytometry. The two kinetics are statistical significantly different (4 °C versus 37 °C) (*p* < 0.05, one-way ANOVA followed by Dunnett’s multiple comparison test). (B) Representative histogram of the increase in peptide uptake at 4 and 37 °C. Only those concentrations where there was a significant difference between the concentrations at the same temperature are represented graphically (*p* < 0.05, unpaired *t* test).

As shown in Figure 1, the GK-1 peptide was internalized by BM-DCs in a dose-dependent manner and more efficiently at 37 °C than at 4 °C (Figure 1A). GK-1 uptake is more gradual at 4 °C than at 37 °C, with significant concentration differences over a wider range (Figure 1B).

**Figure 2:**
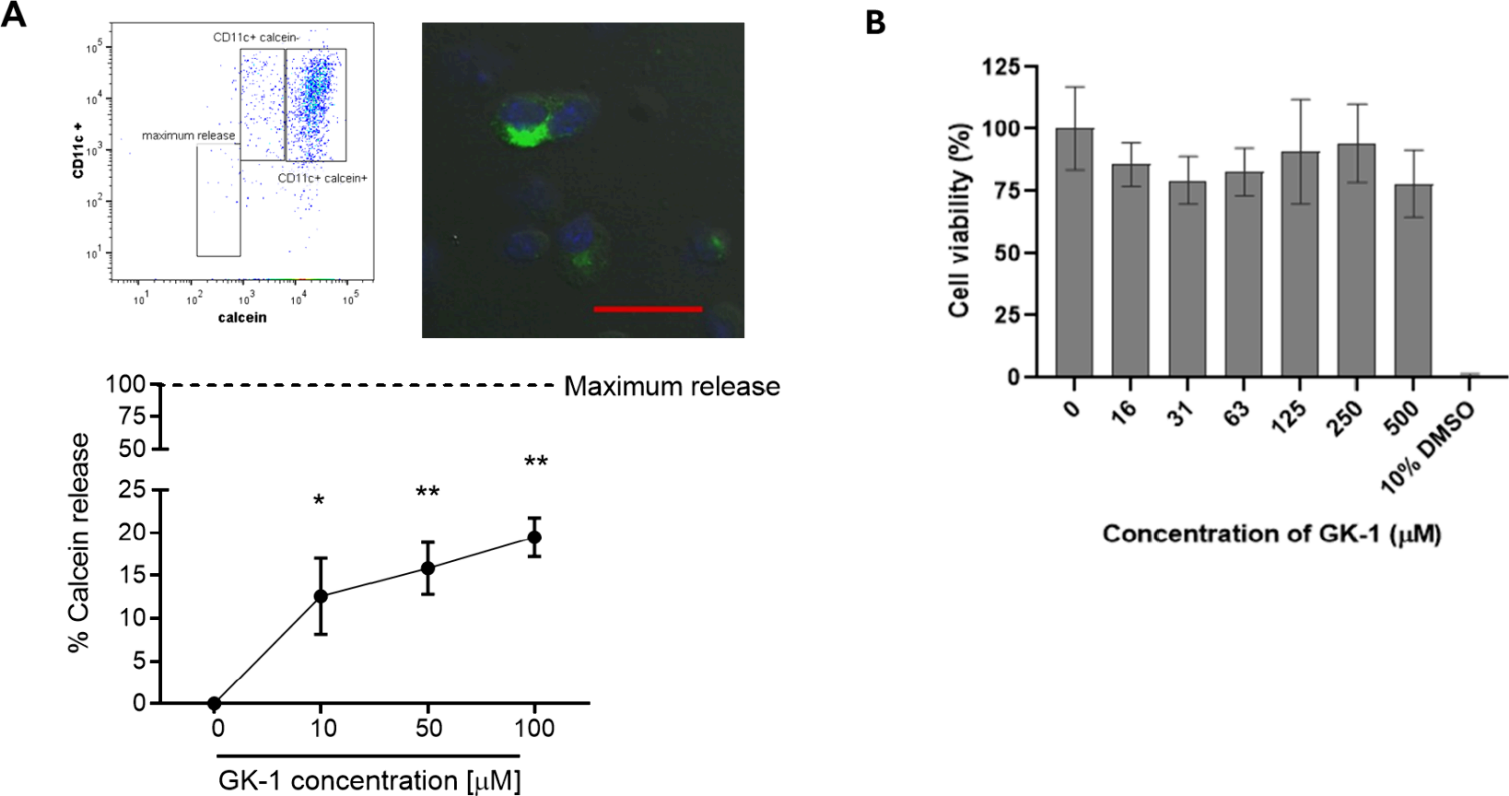
Cytotoxic effects of GK-1 treatment on BM-DC cells. (A) BM-DC cells were stained with calcein AM and treated with different concentrations of the GK-1 peptide (10, 50, or 100 μM). Samples were stained with anti-CD11c antibody and analyzed by flow cytometry. A representative dot plot of the analysis and fluorescence microscopy of the calcein stain (scale bar: 25 μm) are shown in the upper panel. The percentage of calcein release by BM-DC cells was calculated and graphed. *(*p* < 0.05) and **(*p* < 0.01) Statistically significant differences compared to cells treated with medium (unpaired *t* test). (B) BM-DC cell viability as assessed by resazurin assay. The graph indicates the concentration-independent effect on cell viability measured 48 h after the treatment with GK-1 peptide. Each point represents the mean of two independent experiments performed in triplicate. DMSO (10% final concentration in culture medium) was included as a positive control of cytotoxicity.

A possible internalization by transient pore formation mediating direct transport of GK-1 into the cell was tested by calcein acetoxymethyl ester (calcein AM) staining. GK-1-treated BM-DCs (Figure 2A) showed maximum calcein release rates (20%) at the highest concentration of GK-1 (100 μM). This release was not associated with cell death, as shown in a parallel resazurin reduction cytotoxicity assay (Figure 2B), where no cytotoxicity was observed at up to five times higher peptide concentrations (500 μM).

**Figure 3:**
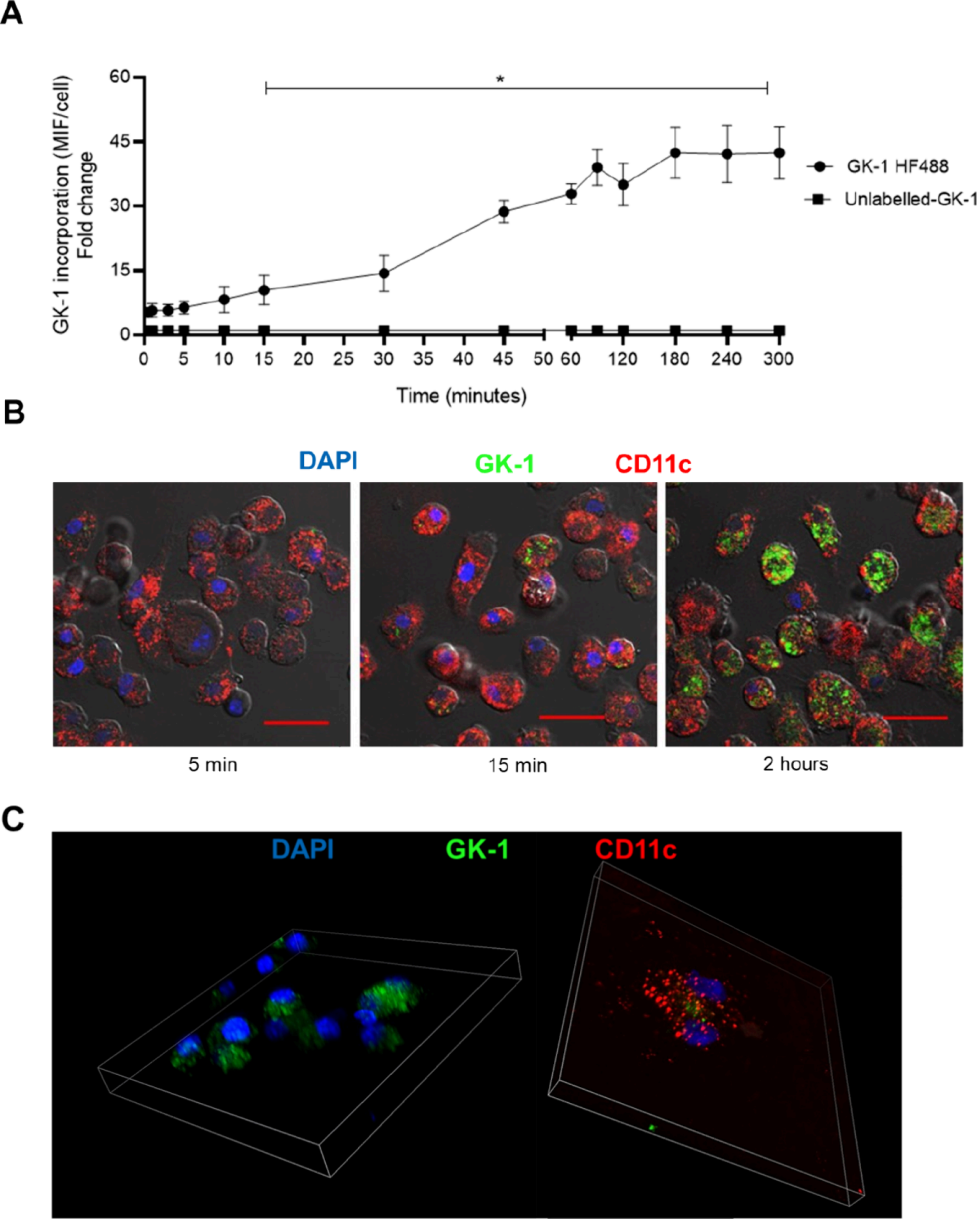
GK-1 uptake is time-dependent. (A) BM-DCs were incubated with GK-1-HF488 (10 μM) for 0.5, 1, 3, 5, 10, 15, 30, 60, 90, 120, 180, 240, or 300 min. Cells were stained with anti-CD11c-PECy5.5 and analyzed by flow cytometry. * Significant differences in peptide uptake analyzed by one-way ANOVA followed by unpaired Student’s *t*-test with respect to baseline time (*p* < 0.05). (B) BM-DCs were cultured in four-well chamber slides and incubated with 10 μM GK-1-HF488 (green) for 5, 15, or 120 min, stained with CD11c Texas Red (red), and processed in mounting medium with DAPI (blue) for confocal fluorescence microscopy. A representative image is shown at 40×. Scale bars are 25 μm. (C) Representative Z-stack images at 60×.

On the other hand, the faster peptide uptake at 37 °C suggested that it may also occur via an energy-dependent process. At 37 °C, GK-1 was significantly detected within BM-DCs 15 min after treatment and increased thereafter, reaching a plateau 45 min later (Figure 3A). GK-1 internalization to BM-DCs was confirmed by confocal microscopy (Figure 3B,C).

**Figure 4:**
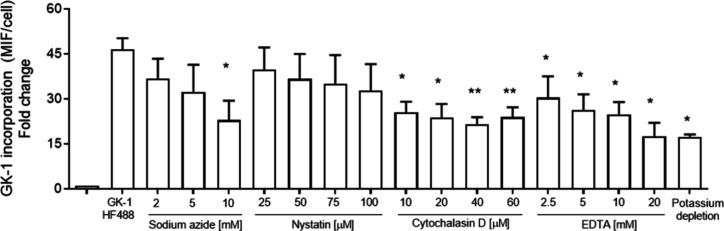
Cellular uptake of GK-1 is inhibited by depletion of cellular ATP, and it is mediated by clathrin endocytosis. BM-DC cells were incubated for 30 min in the presence of either sodium azide (2–10 mM), nystatin (25–100 μM), cytochalasin D (10–60 μM), EDTA (2.5–20 mM), or potassium-depleted buffer in hyperosmotic sucrose solution (450 mM) and thereafter were incubated with 10 μM GK-1 coupled to HiLyte Fluor 488 at 37 °C for 1 h. Samples were trypsinized before staining with anti-CD11c antibody and analyzed by flow cytometry. Statistically significant difference between groups respect group without inhibitor *(*p* < 0.05) and **(*p* < 0.01), by one-way ANOVA and Dunnett’s multiple comparison test.

In parallel experiments, an energy-dependent uptake mechanism was demonstrated by depleting cellular ATP either by preincubating cells with sodium azide or the actin polymerization inhibitor cytochalasin D, or by depleting calcium with EDTA (Figure 4A).

The mechanism underlying GK-1 uptake was also investigated using specific inhibitors of endocytosis pathways. Potassium depletion and a hypertonic environment significantly reduced GK-1 uptake, suggesting that entry into cells occurs via clathrin-mediated endocytosis. In contrast, nystatin, an inhibitor of caveolin-mediated endocytosis, had no effect (Figure 4A). These results suggest that a membrane receptor may be involved in GK-1 cell entry.

### 3.2 *In Silico* Analysis of GK-1 as a Ligand for TLR4/MD2

#### 3.2.1 Molecular Structure of GK-1 and LPS

The electronic and physicochemical properties of GK-1 and LPS were determined by *ab initio* calculations. Initially, five conformations with Pep Fold 3 were predicted from the GK-1 peptide sequence. The lowest energy conformation (−7180.48 AU) was associated with a folded structure characterized by a curve favored by two proline residues (P5 and P8). The folding resulted in a β-sheet, stabilized by hydrogen bonds between G1–P14, Y3–Y12, D7–T9, D7–T10, P14–S17, and Y16–A18; thus, this GK-1 conformation was used for docking studies and MDS. The GK-1 conformation extracted from representative data of the tetrameric ligand–receptor complex in MDS clustering analysis showed an extended conformation capable of further interactions with the receptor and had a total energy of −6988.18 AU. Meanwhile, the LPS conformation obtained from the TLR4-A/MD2-C dimer crystal structure had an *ab initio* total energy of −11 344.65 AU.

**Figure 5:**
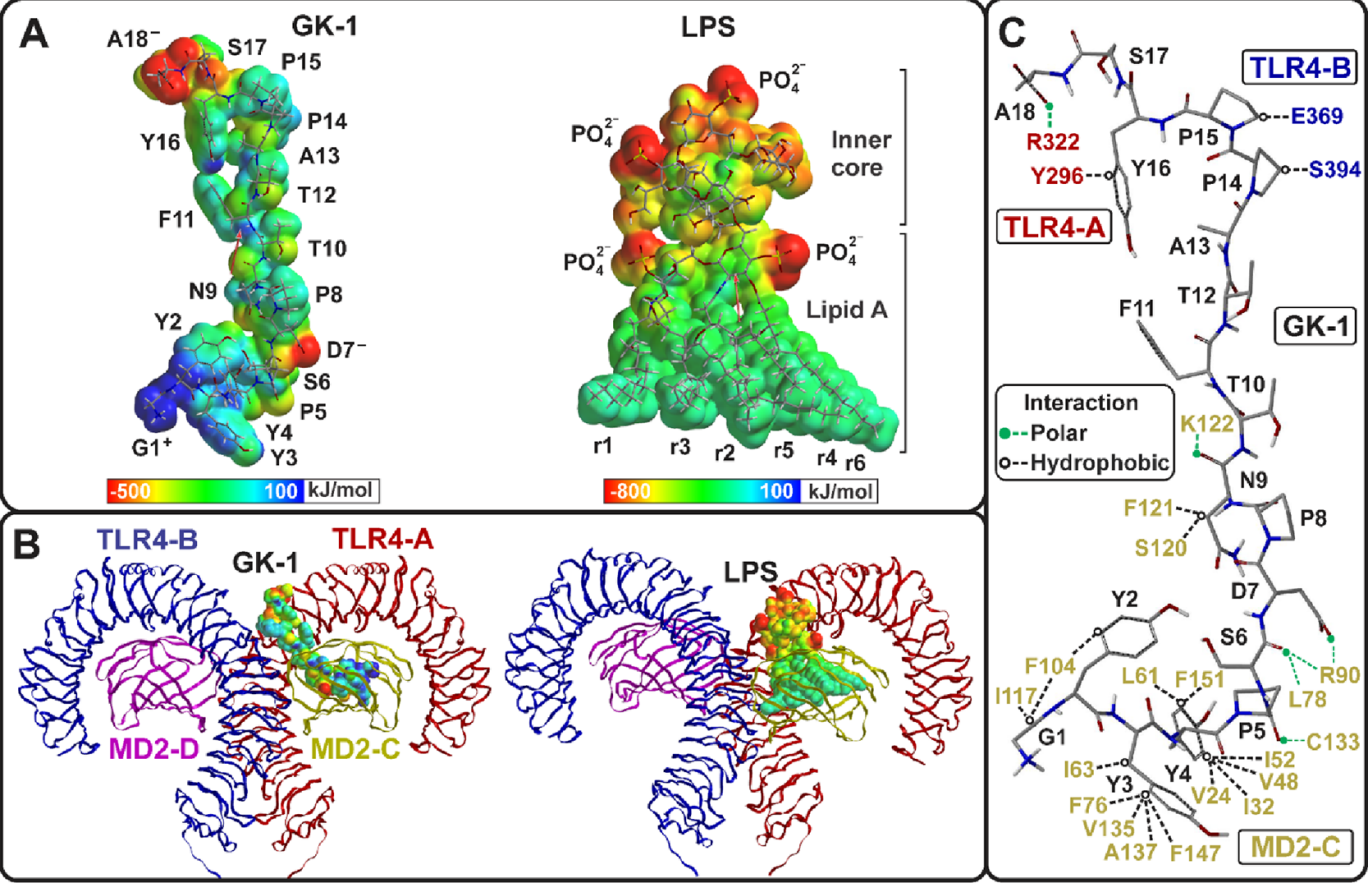
(A) EP and dipole moment (red arrows) of GK-1 and LPS are shown. Negatively charged regions (red) are highlighted in both molecules, due to carboxylate groups (A18 and D7) in GK-1 and phosphate groups in LPS. Positive-neutral regions (blue-green) are due to the amino group of G1, the aromatic rings of Y2, Y3, and Y4 in GK-1, and the lipid chains in LPS. The charge distribution results in a high polarity and produces a large dipole moment oriented in the same direction in both molecules. (B) The electrostatic potential of both ligands in the receptor binding domain is shown. The ligands are preferentially located in the β-cup fold structure, which consists of two antiparallel β-sheets forming the MD2. On the right, the lipid region of LPS occupies the hydrophobic binding pocket (MD2), while GK-1 mimics this feature through the aromatic rings of Y2, Y3, Y4, and P5 and the positively charged amino group of G1. (C) Polar and hydrophobic interactions of GK-1 with residues of TLR4-A/MD2-C and TLR4-B (PDB ID: 3FXI) obtained from MD simulation (only one interaction per residue is shown for clarity).

The electronic configuration calculated under biological conditions (pH 7.2) showed that the molecules have positive and negative formal charges that significantly influence their electronic properties. LPS exhibited an electrostatic potential (EP) pattern with two well-defined phases: a negative (red) phase due to the phosphate groups with negative formal charge −2 located in the inner core and the glucosamines of lipid A, and a neutral (green) phase due to six chains of lipid A. On the other hand, GK-1 had a more heterogeneous EP but retained the two phases. The C-terminus A18 has a negative formal charge that gives rise to a negative EP zone (red), as does the carboxyl of the side chain of D7, and at the amino terminus, the positive charge of G1+ and the aromatic rings of Y2, Y3, Y4, and P5 produce a neutral to positive environment (Figure 5A).

As for the dipole moment, its magnitude indicates that both molecules are polar due to the formal charges, as mentioned above. The dipole vector shows that the polarity is oriented in the same direction in both molecules (Figure 5A). Finally, the larger volume of LPS compared to GK-1 could result in a better fit of the large binding pocket centered in MD2 (Table 1).

**Table 1:** Molecular Properties of GK-1 and LPS^a^

	total energy (AU)	dipole moment (Debye)	polar surface area (Å^2^)	volume (Å^3^)
GK-1	–6988.19	103.41	611.13	1926.60
LPS	–11,344.67	214.35	889.65	2881.21

a Structural comparison of TLR4/MD2 dimers.

Three-dimensional comparison of the X-ray structure of TLR4-A/MD2-C and TLR4-B/MD2-D dimers by superposition and RMSD revealed a high similarity between the two dimers. The RMSD of TLR4-A with TLR4-B was 0.03 Å, and that of MD2-C with MD2-D was 0.02 Å. Based on the overall structural similarity between TLR4-A/MD2-C and TLR4-B/MD2-D, it is likely that their interaction with the binding pocket is similar. The TLR4-A/MD2-C dimer was selected as the target for further *in silico* calculations.

#### 3.2.2 Docking Analysis of GK-1 into the TLR4/MD2 Tetramer

In the docking study, a very large box was used to scan the entire surface of the TLR4-A/MD2-C-TLR4-B/MD2-D tetramer and identify all possible GK-1 docking sites. Nine tetramer-GK-1 complexes were obtained. In all complexes, GK-1 docked to the canonical binding domain as determined by cocrystallization.^21^ The GK-1 tetramer complexes were centered on MD2 of both heterodimers, with four corresponding to TLR4-A/MD2-C and five to TLR4/MD2-D, with conformational variants. This analysis allowed us to exclude accessory binding sites and to place GK-1 in the tetramer for MDS simulation.

#### 3.2.3 MDS of the GK-1-TLR4/MD2 Complex

GK-1 peptide was placed in the same binding site of the LPS ligand to TLR4-A/MD2-C in the best pose according to the docking analysis and used as the input structure for MDS. Clustering analysis of the MDS revealed a high variability in peptide conformation during the first 32 ns. A comparison of the GK-1 atomic backbone with the equilibrium conformation showed differences of more than 10 Å. During this time, the conformation of GK-1 changed from folded to extended. For the remaining 18 ns, GK-1 maintained an extended conformation with the amino terminus in the binding pocket of MD2 and the carboxyl terminus region extending outside of MD2 interacting with TLR4-A and TLR4-B. At this time, the RMSD corresponded to distances of less than 3 Å for peptide–receptor complexes, suggesting that a stable state had been reached.

Peptides are flexible molecules, and their RMSD values are typically much higher as their ensemble is more dynamic. GK-1 reached a stable complex in a relatively short time (32 ns). Proline residues (P5, P8, P14, and P15) may have limited the mobility of the peptide, in addition to the strong ionic interactions associated with GK-1 (carboxylate groups in D7 and A18), resulting in a very stable conformation at 18 ns. The GK-1-TLR4/MD2 complex was stabilized by abundant hydrophobic interactions, primarily in the MD2 binding pocket, and some polar interactions (ionic and hydrogen bonding) distributed across TLR4 and MD2 (Figure 5B).

The hydrophobic environment of the MD2 binding pocket was occupied by aromatic residues (Y2, Y3, Y4, and F11), G1, and N9. However, hydrogen bonding interactions were also observed in MD2, involving the residues S6, T10, and P5 (GK-1), and an ionic interaction between D7 (GK-1) and R90 (MD2). The terminal residues of GK-1 had fewer interactions with TLR4-A and TLR4-B (Table 2).

**Table 2:** Molecular Interactions of GK-1 with the TLR4/MD2 Complex

GK-1 (residue)	TLR4-MD2 (residue)	receptor (monomer)	type of interaction	free energy of interaction (kJ/mol)
Y4	V24	MD2-C	hydrophobic	–65.04
Y4	I32	MD2-C	hydrophobic	
Y4	V48	MD2-C	hydrophobic	
Y4	I52	MD2-C	hydrophobic	
Y4	L61	MD2-C	hydrophobic	
Y3	I63	MD2-C	hydrophobic	–73.00
Y3	F76	MD2-C	hydrophobic	
Y3	V135	MD2-C	hydrophobic	
Y3	A137	MD2-C	hydrophobic	
Y3	F147	MD2-C	hydrophobic	
Y3	F151	MD2-C	hydrophobic	
Y2	F104	MD2-C	hydrophobic	–32.73
Y2	I117	MD2-C	hydrophobic	
Y16	Y296	TLR4-A	hydrophobic	–35.94
T12	K122	MD2-C	H bond	–41.50
T10	K122	MD2-C	H bond	–40.22
S6	L78	MD2-C	H bond	–83.82
S6	R90	MD2-C	H bond	
S17	R322	TLR4-A	H bond	–4.50
P8	R90	MD2-C	hydrophobic	–6.44
P5	C133	MD2-C	H bond	–23.75
P15	E369	TLR4-B	hydrophobic	–45.74
P14	S394	TLR4-B	hydrophobic	–28.51
N9	F121	MD2-C	hydrophobic	–16.83
G1	F104	MD2-C	hydrophobic	–41.48
G1	I117	MD2-C	hydrophobic	
F11	S120	MD2-C	hydrophobic	–60.45
F11	K122	MD2-C	hydrophobic	
D7	R90	MD2-C	ionic	–447.11
A18	R322	TLR4-A	ionic	–462.20
			total	–1 509.26

There was a strong ionic interaction between the carboxyl terminus of A18 (GK-1) and R322 (TLR4-A), and two hydrophobic interactions between P14 and P15 (GK-1) and TLR4-B (Figure 5C and Table 2).

#### 3.2.4 Crystal Structure of the TLR4/MD2-LPS Complex

In the crystal structure of the TLR4/MD2-LPS complex,^16^ each LPS molecule interacts with MD2-C, TLR4-A, and TLR4-B. This complex was used as a reference and compared to the GK-1 receptor complex modeled by MDS. GK-1 and LPS in complex with MD2/TLR4 exhibited hydrophilic (polar) and hydrophobic (van der Waals) interactions, determined by considering cutoff distances of 3.4 and 4.5 Å, respectively (Figure 5C). A number of binding residues were identified, all of which belonged to MD2-C, TLR4-A, and TLR4-B (Table 2). Three types of interactions stabilized the association in the LPS-receptor complex. Abundant hydrophobic interactions, hydrogen bonds, and strong interactions attributed to formal charges were found, as reported previously.^21^ The hydrophobic environment of the MD2 binding pocket allowed the interaction with the LPS lipid chain. Five acyl chains (r2, r3, r4, r5, and r6) of LPS were completely embedded in the MD2 pocket, and the sixth (r1) showed only partial interaction with MD2. In addition, two polar interactions were found at the LPS-MD2 interface: a hydrogen bond with S120 and an ionic interaction with K122 (Figure 5C and Table 3). In LPS, the r1 chain, which is partially exposed outside the MD2 pocket, allowed hydrophobic interactions with TLR4-B. Polar interactions also occurred between TLR4-A and LPS. Hydrogen bonds were formed between D294 and Y296, in addition to an ionic interaction between R322 and K341. Finally, TLR4-B formed only hydrogen bonds (Table 3).

**Table 3:** Molecular Interactions of LPS with Residues of the TLR4/MD2 Complex

LPS	TLR4-MD2 (residue)	receptor (monomer)	type of Interaction
lipid A	R264	TLR4-A	ionic
inner core	D294	TLR4-A	H bond
inner core	Y296	TLR4-A	H bond
inner core	R322	TLR4-A	ionic
inner core	K341	TLR4-A	ionic
inner core	E369	TLR4-B	H bond
lipid A	Q436	TLR4-B	H bond
lipid A	F440	TLR4-B	H bond
lipid A	V24	MD2-C	hydrophobic
lipid A	I32	MD2-C	hydrophobic
lipid A	I46	MD2-C	hydrophobic
lipid A	V48	MD2-C	hydrophobic
lipid A	I52	MD2-C	hydrophobic
lipid A	L54	MD2-C	hydrophobic
lipid A	K58	MD2-C	ionic
lipid A	L61	MD2-C	hydrophobic
lipid A	I63	MD2-C	hydrophobic
lipid A	Y65	MD2-C	hydrophobic
lipid A	L71	MD2-C	hydrophobic
lipid A	F76	MD2-C	hydrophobic
lipid A	L78	MD2-C	hydrophobic
lipid A	V82	MD2-C	hydrophobic
lipid A	L87	MD2-C	hydrophobic
lipid A	R90	MD2-C	hydrophobic
lipid A	R90	MD2-C	hydrophobic
lipid A	E92	MD2-C	hydrophobic
lipid A	I94	MD2-C	hydrophobic
lipid A	F104	MD2-C	hydrophobic
lipid A	I117	MD2-C	hydrophobic
lipid A	F119	MD2-C	hydrophobic
lipid A	S120	MD2-C	H bond
lipid A	F121	MD2-C	hydrophobic
lipid A	K122	MD2-C	ionic
lipid A	I124	MD2-C	hydrophobic
lipid A	F126	MD2-C	hydrophobic
lipid A	C133	MD2-C	hydrophobic
lipid A	V135	MD2-C	hydrophobic
lipid A	F147	MD2-C	hydrophobic
lipid A	F151	MD2-C	hydrophobic
lipid A	I153	MD2-C	hydrophobic

#### 3.2.5 Interaction Energy of the TLR4/MD2-GK-1 Complex

Once the representative conformation of the GK-1 receptor complex was determined by MDS clustering analysis, the interaction free energy was calculated by *ab initio* methods to obtain more accurate values than those estimated by MDS. The interaction free energy was calculated individually for each interacting residue of the receptor (Figure 5B). The total energies of the ligand–receptor complexes and those of the different components (MD2-C, TLR4-A, and TLR4-B) were calculated additively. The interaction of residues G1–F11 of GK-1 with the pocket of MD2-C yielded a free energy of −950.29 kJ/mol. Residues P14–P15 of GK-1 interacted with TLR4-B with a free energy of −34.18 kJ/mol, and the C-terminus of GK-1 interacted with residues Y296 and R322 (TLR4-A) with a free energy of −514.97 kJ/mol. Finally, the total contribution of these interactions to the free energy was −1509.26 kJ/mol.

#### 3.2.6 Interaction Energy of TLR4/MD2-LPS Complexes

In the receptor–LPS complex, the interaction of LPS acyl chains in the MD2 pocket contributed −2227.50 kJ/mol, while TLR4-B contributed −63.74 kJ/mol and TLR4-A contributed −2223.24 kJ/mol. The total free energy contribution of these interactions was −4314.43 kJ/mol.

### 3.3 BM-DCs from C3H/HeJ Mice Are Not Activated by GK-1

**Figure 6:**
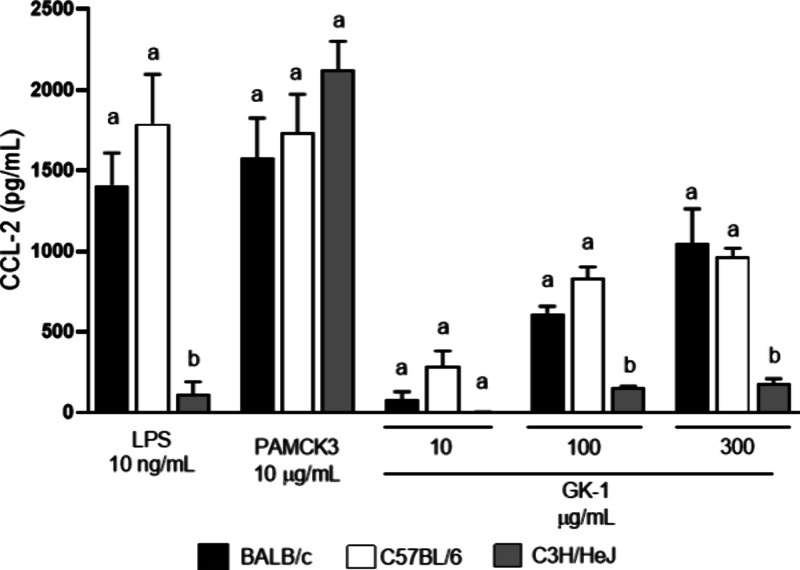
GK-1 mediates BM-DC activation through TLR4 interaction. BM-DCs from BALB/c, C57BL/6, and C3H/HeJ mice were stimulated with either 10 ng/mL LPS, 10 μg/mL Pam_3_CSK_4_, or GK-1 at concentrations of 10, 100, or 300 μg/mL for 24 h. Levels (mean ± SEM) of CCL2 production are shown. Different letters indicate statistically significant differences (*p* < 0.05) between mouse strains for each stimulus. The normality of the data was tested using the Shapiro–Wilk test. Data were compared using one-way ANOVA followed by Tukey’s multiple comparison test.

Additional experiments were performed to confirm that GK-1 mediates BM-DC activation through TLR4. BM-DCs from C3H-HeJ, BALB/c, and C57BL/6J mice were stimulated with GK-1 at concentrations of 10, 100, or 300 μg/mL, with 10 ng/mL LPS as a positive control for TLR4 stimulation, or with 10 μg/mL of the cellular activator Pam3CSK4, which is not a TLR4 agonist. CCL2 levels in the supernatants were measured 24 h later (Figure 6). As expected, little or no CCL2 production was observed in BM-DCs from C3H/HeJ mice exposed to LPS, but a clear response was observed in BM-DCs from BALB/c or C57BL/6 mice. In addition, low or no CCL2 production was observed in cells from C3H/HeJ mice compared to cells from BALB/c or C57BL/6J mice stimulated with GK-1, confirming TLR4-dependent GK-1 activation.

### 3.4 BM-DCs Are Activated by GK-1 through TLR4

**Figure 7:**
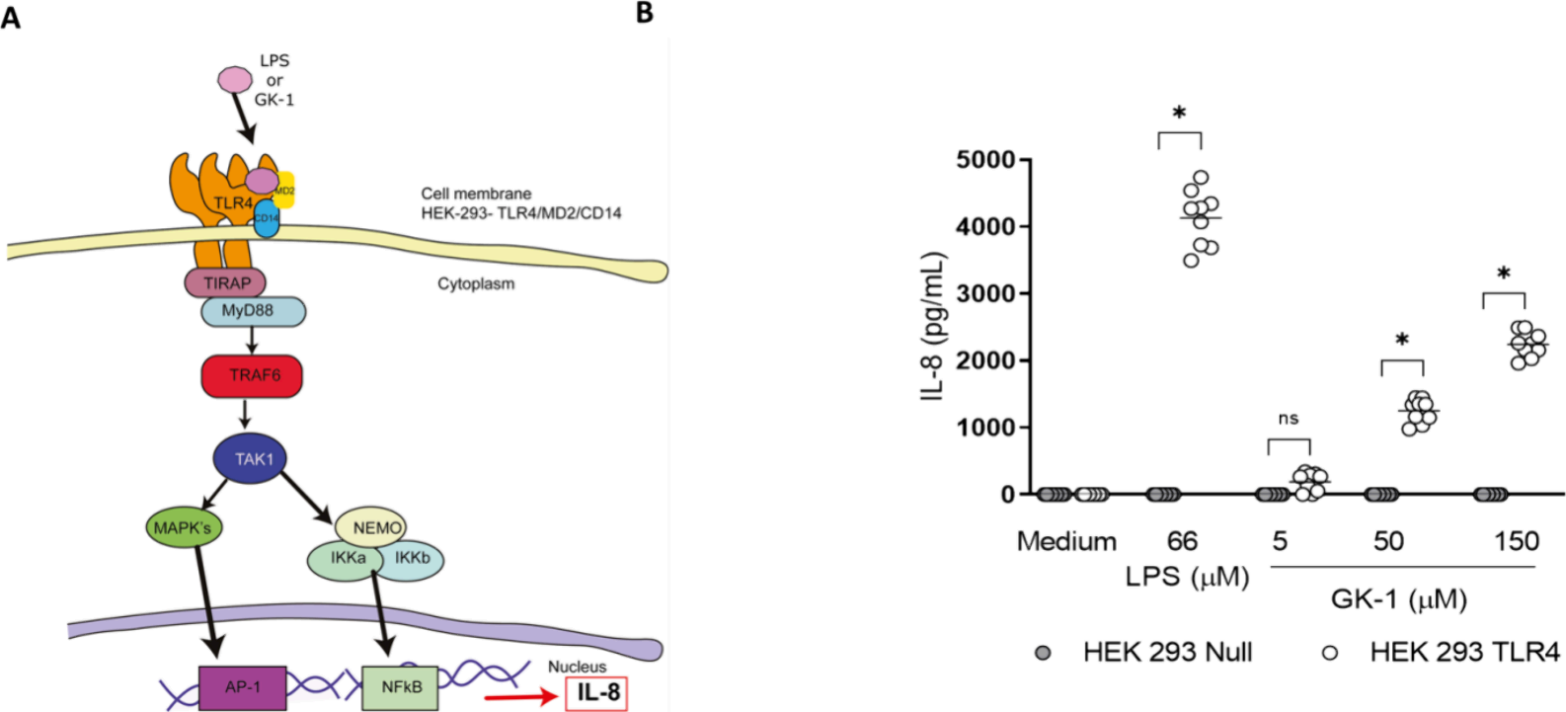
GK-1 mediates the activation of HEK293 cells transfected with the TLR4/MD2/CD14 complex. (A) Representative scheme of HEK293-TLR4 cell activation. TLR4 dimerizes on the membrane by interacting with MD2 and CD14. Once dimerized and upon ligand binding (LPS or GK-1), the canonical TLR4 activation pathway is initiated. This involves the interaction of MyD88 and TIRAP, leading to TRAF6 activation and TAK1 phosphorylation. TAK1 can activate the NEMO complex, resulting in the translocation of NF-κB. NF-κB activation leads to the production of proinflammatory cytokines, including IL-8, which can be used as a reporter of TLR activation. (B) HEK-294 null or TLR4 complex-transfected HEK293 cells were stimulated with 100 ng/mL LPS or GK-1 at concentrations of 5, 50, or 150 μM for 16 h. Mean IL-8 secretion levels are shown of three independent experiments. Data were analyzed by two-way ANOVA followed by Sidak’s multiple comparison. *p < 0.05 was considered significant. The illustration was made by the authors using Adobe Illustrator software 24.0.1

MyD88-dependent receptors mediate the activation of APCs by GK-1.^11^ Therefore, the possible involvement of different types of TLRs in immune cell activation was evaluated experimentally using HEK293-null and transfected HEK293 cells. GK-1 did not interact with TLR1, TLR2, TLR3, TLR5, TLR6, or TLR7 (Figure S1). Evidence of GK-1 activation was observed in HEK293 cells transfected with TLR4 (Figure 7A). Cultures of HEK293-hTLR4 cells were exposed to GK-1 at concentrations ranging from 10 to 300 μg/mL or to 100 ng/mL LPS. As shown in Figure 7B, GK-1 activated HEK293-hTLR4 cells in a dose-dependent manner as evidenced by the expression of the reporter cytokine IL-8. No detectable levels of IL-8 were found in the supernatants of HEK293-null cells exposed to GK-1 (Figure 7B).

**Figure 8:**
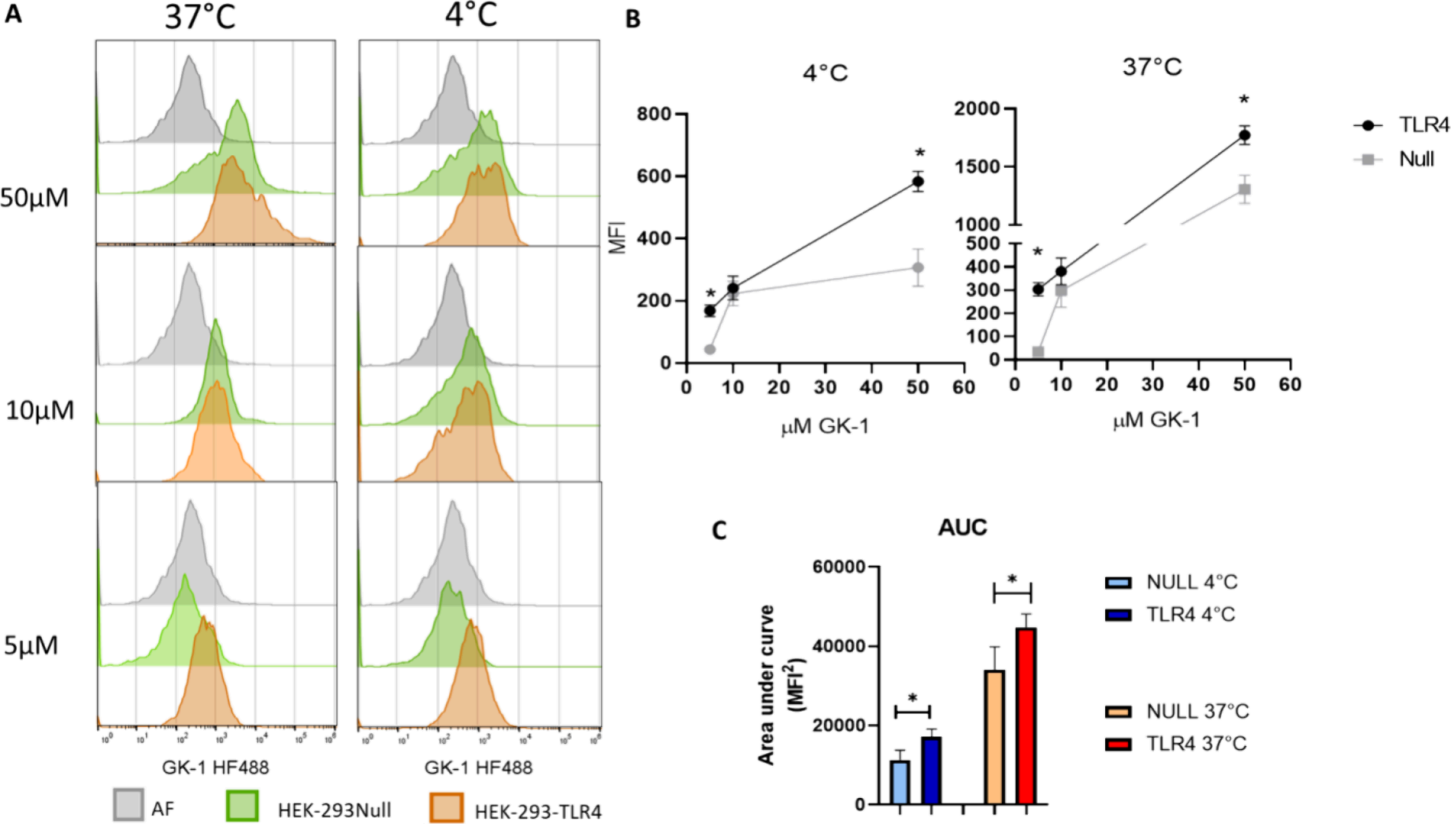
TLR4 promotes GK-1 entry to the cell. (A) Representative histograms of GK-1-HF-488 uptake. Cells not exposed to GK1-HF488 (gray) represent cell autofluorescence. HEK293-null cells (green) and HEK293-TLR4 cells (orange) are shown. (B) Mean fluorescence intensity (MFI) of cells treated with GK1-HF488 at concentrations ranging from 5 to 50 μM. The black line shows the MFI of HEK293-TLR4 cells, and the gray line shows the MFI of HEK293-null cells. (C) Area under the curve for each group of cells showing that GK-1-HF488 uptake is higher in TLR4-expressing cells and the difference is greater at 37 °C. GK-1-HF488 internalization levels were compared by two-way ANOVA. Sidak multiple comparisons were used to compare the groups. Data are expressed as mean ± SEM, *p < 0.05.

The presence of GK-1-HF488 was detected in HEK293-null and TLR4-HEK293 cells transfected at 4 and 37 °C. To determine whether GK-1 uptake depends on its interaction with the TLR4/MD2 complex, the presence of the peptide was examined in HEK293-null and TLR4-HEK293-transfected cells, at 4 and 37 °C. As shown in Figure 8, TLR4 promotes GK-1 uptake, especially in cells exposed to 5 μM GK-1. At 4 °C, only HEK293-TLR4 cells internalized GK-1-HF488, showing fivefold higher MFI values than HEK293-null cells. A similar trend was observed for GK-1 internalization at 37 °C, with MFI values up to 30-fold higher in transfected cells than in HEK293-null cells. This points out the relevance of TLR4 in the uptake and cellular activation of GK-1.

## 4 Discussion

Previous studies have shown that GK-1 increases the expression of the activation molecules CD80 and CD86 on LPS-pulsed APCs, with subsequent production of effector molecules, including the chemokines CCL2 and CCL3, and the proinflammatory cytokines IL-6 and TNF-α.^12,41^ These effects were found to be mediated by phosphorylation of MAPKs p38, JNK, and ERK 1/2.^11^ Furthermore, a significant reduction in NF-κB-p65 translocation to the nucleus was observed in BM-DCs from MyD88 KO mice stimulated with GK-1 *in vitro*. In addition, transcriptional profiling of GK-1-treated Mφ revealed overexpression of several molecules involved in TLR signaling pathways.^12^ Taken together, these results suggest the involvement of a TLR as a GK-1 receptor.

Evidence in this manuscript shows that both energy-independent and energy-dependent processes mediate GK-1 entry (Figure 1). Direct membrane diffusion may be favored by its small size and hydrophobicity.^42^ This effect may be mediated by direct translocation of the peptide into the cell interior through the formation of transient pores that do not compromise membrane integrity as occurs with other small peptides^43^ (Figure 2B).

The cytotoxicity study conducted in parallel to the quantification of calcein release demonstrated that the increased membrane permeability to calcein after GK-1 exposure was not due to cell death or toxicity, since no evidence of cytotoxicity was observed at any of the tested concentrations. It is important to notice that such high concentrations (up to 500 μM) were only used for experimental purposes to prove that GK-1 was not harmful to BM-DC in cultures. *In vivo* antitumor and antimetastatic effects in mouse models are observed at doses as low as 0.5–5 mg/kg, which result in plasma concentrations significantly lower than those tested in the cytotoxicity assay.^4−6,44,45^

Entry by an energy-dependent mechanism may involve endocytic uptake through a membrane receptor. Our results support GK-1 entry via clathrin-mediated endocytosis, as its uptake is reduced by (1) ATP depletion by sodium azide;^17^ (2) cytochalasin D-induced depolymerization of actin filaments necessary for endocytic processes;^19^ and (3) calcium sequestration by EDTA.^18,42^ In addition, by depleting potassium ions, GK-1 was found to enter cells via a clathrin-dependent endocytosis process, as potassium ions prevent the formation of clathrin networks associated with the inner layer of the plasma membrane.^43,44^ Furthermore, the use of nystatin, an inhibitor of caveolin-mediated endocytosis,^18^ did not affect GK-1 entrance (Figure 4). Taken together, these findings support that GK-1 can also enter cells via a receptor through a clathrin-dependent process, which may play a role in the modulatory effect of GK-1 on the host immune response, as has been reported for other small peptides.^46,47^

Given the above findings, a TLR-mediated entry mechanism is a likely candidate for underlaying this endocytic process. To determine the involvement of a TLR, GK-1 activation of HEK cells transfected with TLR2/6, TLR3, TLR4, TLR5, and TLR7 or nontransfected (null cells) was assayed with GK-1. Our results support that GK-1 activation is mediated by TLR4 (Figure 7 and Figure S1), a receptor that also facilitates the uptake of GK-1 (Figure 8). Entry of small molecules into the cell can occur by a variety of mechanisms, some of which are energy dependent, involving changes in the cytoskeleton, while others are dependent on concentration gradients, as in the case of channels or transient pore formation.^48^ Our results suggest that the main mechanism of GK-1 entry into the cell is clathrin-mediated endocytosis (Figure 4). This is consistent with the mechanisms of endocytosis described for TLR4, which are dependent on dynamin and clathrin.^49^ By binding to TLR4, the peptide could be internalized more efficiently, as shown in Figure 8. It would be interesting to investigate whether GK-1 can activate an alternative TLR4 pathway through the production of type I interferons.^50,51^ In contrast, LPS, a potent ligand of TLR4, enters into the cells through a TLR4-independent pathway involving CD14.^47^

Endocytosis processes are known to be inhibited at temperatures below 10 °C,^52^ which is consistent with the threefold reduction in GK-1 uptake at 4 °C in TLR4-transfected cells compared to cells incubated at 37 °C. It should be noted that GK-1 entry is not solely dependent on endocytosis, as the peptide can enter cells at 4 °C. Thus, GK-1 could also enter cells by a concentration-dependent mechanism, following Fick’s law,^53^ independent of TLR receptor expression.

Docking and molecular dynamics studies supported these results and provided additional evidence that warrants further discussion (Figure 7). *In silico* analysis of the potential interaction of GK-1 in the TLR4/MD2 heterodimeric complex shows that GK-1 can stably occupy the ligand-binding domain. The negative formal charges of LPS and GK-1 strongly influence their electronic properties and thus receptor binding. The EP shown by LPS with a well-defined biphasic pattern was also observed in GK-1, but to a lesser extent (Figure 5). The neutral-positive phase of EP in LPS was due to the lipid chains that associated deeply and completely occupying the hydrophobic pocket of MD2. Thus, the abundant hydrophobic interactions of the lipid chains contributed almost half (−2227.50 kJ/mol) of the total binding energy of the LPS-TLR4/MD2 complex. In GK-1, aromatic residues (Y2, Y3, Y4, and F11) buried deep in the MD2 binding pocket contributed to hydrophobic interactions and bulk, and some polar interactions also enhanced receptor binding. In particular, the ionic interaction between D7 (GK-1) and R90 (MD2), which functions as an anchor, stabilizing the adopted conformation of the GK-1 peptide,^54−56^ led to an especially high interaction energy with MD2 (−950.29 kJ/mol). On the other hand, the negative phase of the EP in LPS due to the polar groups (OH and PO_4_^-2^), allowed the binding with polar residues of TLR4-A and TLR4-B, their approach and association with MD2. Despite the lower number of polar interactions of LPS compared to those shown in MD2, polar interactions gave rise to strong associations, in particular through ionic interactions. Thus, the binding energy was especially high in the interaction with TLR4-A (−2223.24 kJ/mol) and very small with TLR4-B (−63.74 kJ/mol). GK-1 had a lower number of interactions with TLR4-A and TLR4-B compared to LPS. However, a strong ionic interaction of A18 (GK-1) with R322 (TLR4-A) stands out for its evident contribution to the binding free energy (−514.97 kJ/mol) supporting the possibility of attraction and binding with TLR4. Furthermore, the pattern shown in LPS is preserved, with respect to TLR4-B, with a minimal contribution (−34.18 kJ/mol).

The binding pattern of GK-1 to the TLR4/MD2 complex matches some features displayed by LPS in terms of structure and binding energy, indicating that it can occupy the TLR4/MD2 complex and initiate biological responses. The total interaction free energies of LPS (−4314.43 kJ/mol) and GK-1 (−1509.26 kJ/mol) indicates a clearly higher affinity of LPS compared to GK-1 to initiate signaling. Further studies of the colocalization of both TLR4 and GK-1 could allow the confirmation that GK-1 is effectively a ligand of TLR4.

The activation of cells by the interaction of LPS with TLR4 may induce an overstimulation of the inflammatory response, leading to chronic inflammation in noninfectious diseases such as atherosclerosis, rheumatoid arthritis, and inflammatory bowel disease.^57^ It is possible that GK-1, a ligand with 2.8 lower affinity for TLR4, may also trigger inflammation but with a less sustained response that enhance the specific Th1 immunity to specific antigen when coadministered with a certain antigen, as it was the case of the influenza vaccine,^41^ or tumoral antigens when administered to mice carrying an experimental breast cancer.^6^ Moreover, GK-1 immunotherapy has been found that can also control other types of cancers that overexpress TLR4 such as the murine model of melanoma.^4^

Overall, the demonstrated preclinical antitumor activity and safety of GK-1, together with its low cost compared with immunotherapeutic approaches, support its potential as a new alternative of potential usefulness in cancer immunotherapy.

## 5 Conclusions

Overall, our results strongly suggest that GK-1 exerts its immunomodulatory properties employing the TLR4 receptor; however, unlike LPS, it promotes a more subtle inflammatory response, which may be beneficial through improving antigen presentation, restoring antitumor immunity and the consequent control of tumor growth and metastasis.

## Supporting Information Available

Evaluation of cellular activation mediated by GK-1 in HEK-294 null or TLR2/6, TLR5, TLR3, and TLR7 complex-transfected HEK-294 cells (PDF)

## Abbreviations

ANOVA: analysis of variance
APCs: antigen-presenting cells
BM-DCs: bone marrow-derived dendritic cells
calcein AM: calcein acetoxymethyl ester
CCL2: CC chemokine ligand 2
CCL3: CC chemokine ligand 3
DAMPs: damage-associated molecular patterns
DAPI: 4′,6-diamidino-2-phenylindole
DCs: dendritic cells
DFT: density functional theory
DMEM: Dulbecco’s modified Eagle medium
EDTA: ethylenediaminetetraacetic acid
ELISA: enzyme-linked immunosorbent assay
EP: electrostatic potential
ERK: extracellular signal-regulated kinase
FBS: fetal bovine serum
GM-CSF: granulocyte-macrophage colony-stimulating factor
HEPES: 2-hydroxyethylpiperazine-1-ethanesulfonic acid
IFN-γ: interferon gamma
IL-6: interleukin 6
IL-8: interleukin 8
IRF: interferon regulatory factor
JNK: c-Jun N-terminal kinase
LINCS: linear constraint solver
LPS: lipopolysaccharide
MAPK: mitogen-activated protein kinase
MDS: molecular dynamics simulation
Mφ: macrophages
MFI: mean fluorescence intensity
MHC: major histocompatibility complex
MyD88: myeloid differentiation primary response 88
NEMO: NF-κB essential modulator
NF-κB: nuclear factor kappa B
NVT: number of particles, volume, and temperature
NPT: number of particles, pressure, and temperature
PAMPS: pathogen-associated molecular patterns
PBS: phosphate-buffered saline
PD-1: programmed cell death-1
RMSD: root mean square deviation
RPMI 1640: Roswell Park Memorial Institute 1640 culture medium
SEM: standard error of the mean
TAK1: transforming growth factor beta activated kinase 1
TIRAP: Toll-interleukin 1 receptor adaptor protein
TNFα: tumor necrosis factor alpha
TLR1, TLR2, TLR3, TLR4, TLR5, TLR6 and TLR7: tool-like receptors 1, 2, 3, 4, 5, 6, and 7
TRAF6: TNF receptor-associated factor 6
