# Supplementary material for: Exploring the Mechanisms Underlying Cellular Uptake and Activation of Dendritic Cells by the GK-1 Peptide
Source: ACS Omega. 2024 Nov 28;9(50):49625–38. doi: 10.1021/acsomega.4c07736 (PMC11656211; doi:10.1021/acsomega.4c07736)
Supplement: Supplementary file 1 — ao4c07736_si_001.pdf [file ao4c07736_si_001.pdf]

Exploring the mechanisms underlying cellular uptake and activation of dendritic cells by the GK-1 peptide

Jacquelynne Cervantes-Torres[1,2]‡, Juan A. Hernández-Aceves[1]‡, Julián A. Gajón Martínez[3], Diego Moctezuma Rocha[1], Ricardo A. Vázquez Ramírez[4], Sergio Sifontes-Rodríguez[5], Gemma L. Ramírez-Salinas[1,6], Luis A. Mendoza Sierra[4], Laura Bonifaz Alfonzo[3], Edda Sciutto[1], Gladis Fragoso[1]**

1- Departamento de Inmunología, Instituto de Investigaciones Biomédicas; Universidad Nacional Autónoma de México, Ciudad de México, MX 04510
2- Departamento de Microbiología e Inmunología, Facultad de Medicina Veterinaria y Zootecnia, Universidad Nacional Autónoma de México, Sede Circuito Escolar Edificio A 1er Piso, Ciudad de México, MX 04510
3- Unidad de Investigación Médica en Inmunoquímica, Hospital de Especialidades, CMN Siglo XXI, Instituto Mexicano del Seguro Social, Ciudad de México, MX 06600
4- Departamento de Biología Molecular y Biotecnología, Instituto de Investigaciones Biomédicas, Universidad Nacional Autónoma de México, Sede Tercer Circuito Exterior Edificio C 1er Piso, C-146, Ciudad de México, MX 04510
5- Investigador por México del CONAHCyT adscrito al Departamento de Inmunología, Instituto de Investigaciones Biomédicas. Universidad Nacional Autónoma de México, Sede Circuito Escolar Edificio A 1er Piso, Ciudad de México, MX 04510

6- Present address: Escuela Superior de Medicina, Instituto Politécnico Nacional, Calle Plan de San Luis y Díaz Mirón s/n, Miguel Hidalgo, Casco de Santo Tomás. Ciudad de México, MX 11340

† These authors contributed equally to this study.

*Corresponding authors: Departamento de Inmunología, Instituto de Investigaciones Biomédicas, Universidad Nacional Autonoma de Mexico. Sede Circuito Escolar Edificio A 1er Piso / Oficina 116, Mexico City, 04510, Mexico. Phone: +52 55562-29250 ext. 44104. E-mails: gladis@unam.mx, edda@unam.mx,

Evaluation of the effect of GK-1 on HEK-293 cells expressing different TLRs

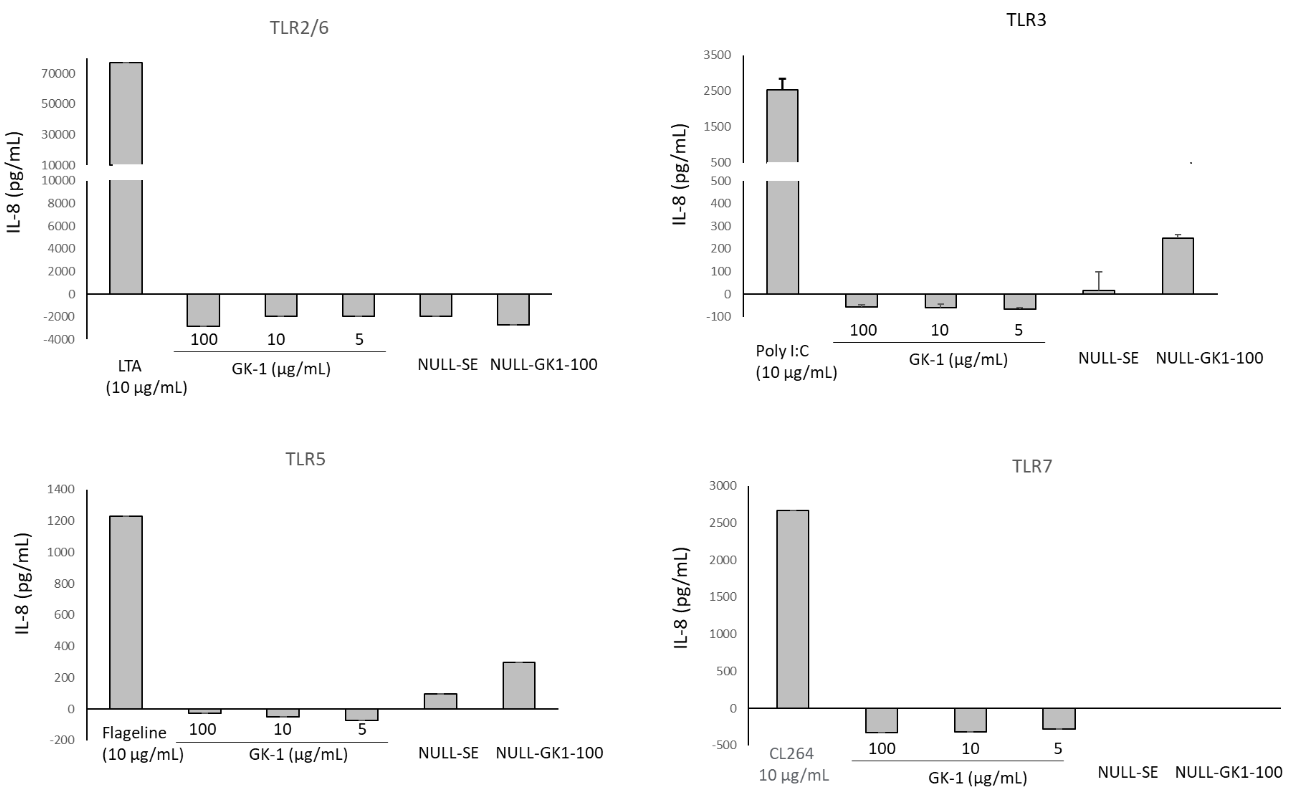

Supplementary Figure S1. **GK-1 does not produce IL-8 in cells HEK-293 transfected with TLR2/TLR6, TLR3, TLR5 or TLR7**. IL-8 quantification in HEK-293 cells expressing either TLR2/TLR6, TLR3, TLR5 or TLR7. Positive controls for each cell line were: Lipoteichoic acid (LTA) Polyinosinic:polycytidylic acid (poly I:C), flageline, and CL264 (InvivoGen® , CA, US).
